# CT reconstruction based 3D model of the digital cushion’s blood supply in the hind foot of an African savanna elephant (*Loxodonta africana*)

**DOI:** 10.3389/fvets.2024.1399392

**Published:** 2024-05-13

**Authors:** László Zoltán Reinitz, Franka Lenzing, Endre Papp, Alexandra Biácsi, Dániel Fajtai, Örs Petneházy

**Affiliations:** ^1^Department of Anatomy and Histology, University of Veterinary Medicine Budapest, Budapest, Hungary; ^2^Nyíregyházi Állatpark Nonprofit Kft. (Sosto Zoo), Nyíregyháza, Hungary; ^3^Medicopus Nonprofit Ltd., Kaposvár, Hungary; ^4^Institute of Animal Physiology and Nutrition, Department of Physiology and Animal Health, Hungarian University of Agriculture and Life Sciences, Kaposvár, Hungary

**Keywords:** African savanna elephant, CT reconstruction, hindfoot, blood supply, anatomy, 3D

## Abstract

**Introduction:**

Foot health is crucial for elephants, as pathological lesions of the feet are a leading cause of euthanasia in captive elephants, which are endangered species. Proper treatment of the feet, particularly in conditions affecting the digits and the digital cushion, requires a thorough understanding of the underlying anatomy. However, only limited literature exists due to the small population and the epidemiological foot diseases which often precludes many deceased elephants from scientific study. The aim of this study was to provide a detailed anatomical description of the blood supply to the African elephant’s hindfoot.

**Methods:**

The healthy right hindlimb of a 19-year-old deceased female African savanna elephant was examined using computed tomography. Following a native sequence, 48 mL of barium-based contrast agent was injected into the caudal and cranial tibial arteries, and a subsequent scan was performed. The images were processed with 3D Slicer software.

**Results:**

The medial and lateral plantar arteries run in a symmetrical pattern. They each have a dorsal and a plantar branch, which reach the plantar skin before turning toward the axial plane of the sole to reach the digital cushion from the proximal direction. An accurate 3D model of the arteries and the bones of the foot, a set of labeled images and an animation of the blood supply have been created for ease of understanding.

**Discussion:**

In contrast to domestic ungulates, the digital cushion of the hindlimb is supplied differently from that of the forelimb. The lack of large vessels in its deeper layers indicates a slow regeneration time. This novel anatomical information may be useful in the planning of surgical interventions and in emergency medical procedures.

## 1 Introduction

Elephants, belonging to the family *Elephantidae* and to the order *Proboscidea*, hold a symbolic place in the annals of human history, representing strength, grace, wisdom, and peace (1). The African savanna elephant (*Loxodonta africana*) is the largest terrestrial animal, with an average shoulder height:body weight ratio of 3.2 m:6000 kg for males and 2.6 m:3000 kg for females (2), and has been classified as endangered on the Red List of the International Union for Conservation of Nature since 2001 (3).

Wild elephants only lie down to sleep for about an hour every 3 to 4 days; otherwise they only sleep in a standing position for 1 to 2 h per day (4). While captive individuals may have slightly different sleeping habits, the legs and feet are critically important to their health. The limbs of the elephant are columnar, and, as in most quadruped animals, the forelimbs carry a greater weight (5–7). The limbs remain columnar during slow-paced movement, with a limited flexion appearing at higher speeds (8). The primary function of the hindlimb is to initiate movement and transmit thrust to the trunk, as in all quadrupeds. However, the elephant hindlimb shares many similarities with the human hindlimb in morphology (e.g., elongated femur) and movement pattern (no significant rotation or abduction/adduction in the stifle) (7, 8). This stiffness of movement, the poor limb configuration (9, 10) an walking on hard surfaces, often in cold, damp conditions with limited space (6, 10, 11) result in widespread occurrence of foot diseases in captive elephants.

Approximately 50% of captive elephants experience foot-related diseases at some point in their lives (12). In a recent paper, only 26% of the United Kingdom’s captive elephant population showed normal locomotion (9). Another study found that only 1.2% of the elephants examined had no visible lesions on their nails and pads (13). Among the varieties of diseases, chronic unresponsive foot infections stand out as a leading cause for euthanasia in captive elephants (14). While certain external lesions such as nail cracks, foreign bodies, and visible infections can be identified without the need for diagnostic imaging (15), these superficial lesions can also lead to more severe diseases, particularly those affecting the underlying bones, such as osteomyelitis, ascending soft tissue infections, and infectious arthritis. These conditions are challenging to diagnose and treat, as the elephants spend most of their time standing; this outlook could however be improved with a better understanding of the underlying anatomical structures (8, 16).

The anatomy of the feet in the different elephant species is remarkably similar despite the significant differences in their habitat (17). Their hindfoot is generally smaller than the forefoot as it bears less weight, with the forelegs bearing up to 60%, and the hindlegs bearing about 40%. Its shape is ovoid in character due to a lateral (17) or mediolateral (18) compression, which is pronounced in African savanna elephants. The African savanna elephant has no phalangeal bones distal to the first metatarsal bone, and the digit is only evidenced by a single, axial sesamoid bone. Smuts and Bezuidenhout (19) excluded this structure from the digits and view the African elephant as having only four digits, whilst Ramsay and Henry (17) accepted and counted this rudiment as the first digit. Digits three and four are the largest and therefore carry the greatest weight.

A significant digital cushion is located behind the slanted digits, under the tarsus. It is composed of fibrous connective tissue containing collagen, reticulin, and elastic fibers. Through the ability to compress and expand during locomotion, the digital cushion can be counted as a dynamic feature which makes the movements of the feet more active than previously assumed (18). The compression and relaxation mechanism work like a pump as the digital cushion facilitates the return of venous blood toward the central venous system against gravity (14), which is a critical function given the size and the constant movement of the elephant. While digital cushions are also described in many other mammalian species, such as horses (20, 21), their significance in locomotion is extraordinary in elephants due to the absence of the weight-bearing suspensory apparatus of the distal phalanx, which is the chief weight-bearing structure in horses (22).

The available literature for the vasculature of the elephant’s hindlimb is scarce. Ramsay and Henry (17), much like Smuts and Bezuidenhout (19), focus on the locomotive system. Ramsay and Henry found that the cranial tibial artery (*arteria (a.) tibialis cranialis*), which provides the dorsal blood supply of the pes in equines and ruminants (21, 23), became small at the level of the tarsus (11). The same paper reported that the caudal tibial artery provided the majority of the plantar blood supply (17); it divided into a medial and a lateral branch at the calcaneus, with the medial branch running through the *canalis tarsi*. Both the medial and the lateral branches were shown to extend into the digital cushion on their respective sides (17, 24), but the paper does not give any further description about the exact location or further branching of these arteries. There are no diagrams or photos available about the foot’s blood supply to improve understanding.

Petneházy et al. described the blood supply of the digital cushion in the forelimb of an Asian elephant using computed tomography (CT)-based reconstruction (25). They found that the metacarpal artery gave rise to a short trunk, which divided into a palmar and a dorsal branch, both of which supply the digital cushion from their respective directions; the short trunk also gave off a branch running toward the first digit. Given the similarities in the foot anatomy between the two species, a similar arrangement of vessels can be hypothesized to exist in the forelimb of the African savanna elephant as well.

To date, externally visible landmarks have been used in surgical procedures (26) to guide incisions. This approach leads to complications in procedures such as phalangeal removal (27), because the course and size of the vessels are unpredictable. In addition, diseases such as osteomyelitis and osteitis are currently treated with antibiotics injected locally into the vessels without knowing their exact course. Such treatment is done by looking for superficial veins with ultrasound (28). A detailed understanding of the systemic circulation and blood supply is of great necessity to form the basis of surgical treatments of the limbs, while avoiding vascular complications, and to effectively deliver drugs locally via specific vessels that supply a targeted tissue area. The aim of this study is to improve the current state of knowledge by providing a detailed description of the blood supply of the digital cushion and the digits in the African elephant, supported by images and 3D animation.

## 2 Materials and methods

The right hindfoot of a deceased African savanna elephant at the Sosto Zoo (Nyíregyházi Állatpark Nonprofit Kft. (Sosto Zoo), HRSz15010/2, Sóstói út, H-4431 Nyíregyháza, Hungary) was used for the study. The 19-year-old female specimen was parent-reared in a peer group and kept on 1 hectare of landscaped forest soil with mounds and ditches. In addition, there was an indoor facility with 400 square meters of floor area covered with synthetic resin. Use of the outdoor area was limited to 6 h per day during winter conditions. The female died of Clostridium enterotoxemia, but her foot was clinically assessed as healthy. The leg was cut at the tibiotarsal joint (*articulatio (art.) tibiotarsale*) with a bone saw and was frozen at −4°C. The leg was then placed in a room temperature environment for 48 h and the cranial and the caudal tibial arteries were cannulated (16G intravenous catheter, B. Braun AG., Melsungen, Germany) before the examination was performed.

Three CT series were produced at the Kaposi Somogy County Teaching Hospital Dr. József Baka Diagnostic, Radiation Oncology, Research and Teaching Center (Kaposvár, Hungary) with a SIEMENS SOMATOM Sensation Cardiac CT (Multislice scanner, Siemens AG, Erlangen, Germany). A native series was taken, resulting in 2144 slices (Exposure Time: 16.04 s; Scanning Length: 493 mm; Exposed Range: 464 mm; Nominal Single Collimation Width: 0.6 mm; Nominal Total Collimation Width: 38.4 mm; Pitch Factor: 0.8 ratio; Number of X-Ray Sources: 1; KVP: 120 kV; Maximum X-Ray Tube Current: 240 mA; X-Ray Tube Current: 239 mA; Exposure Time per Rotation: 1 s).

**Figure 1:**
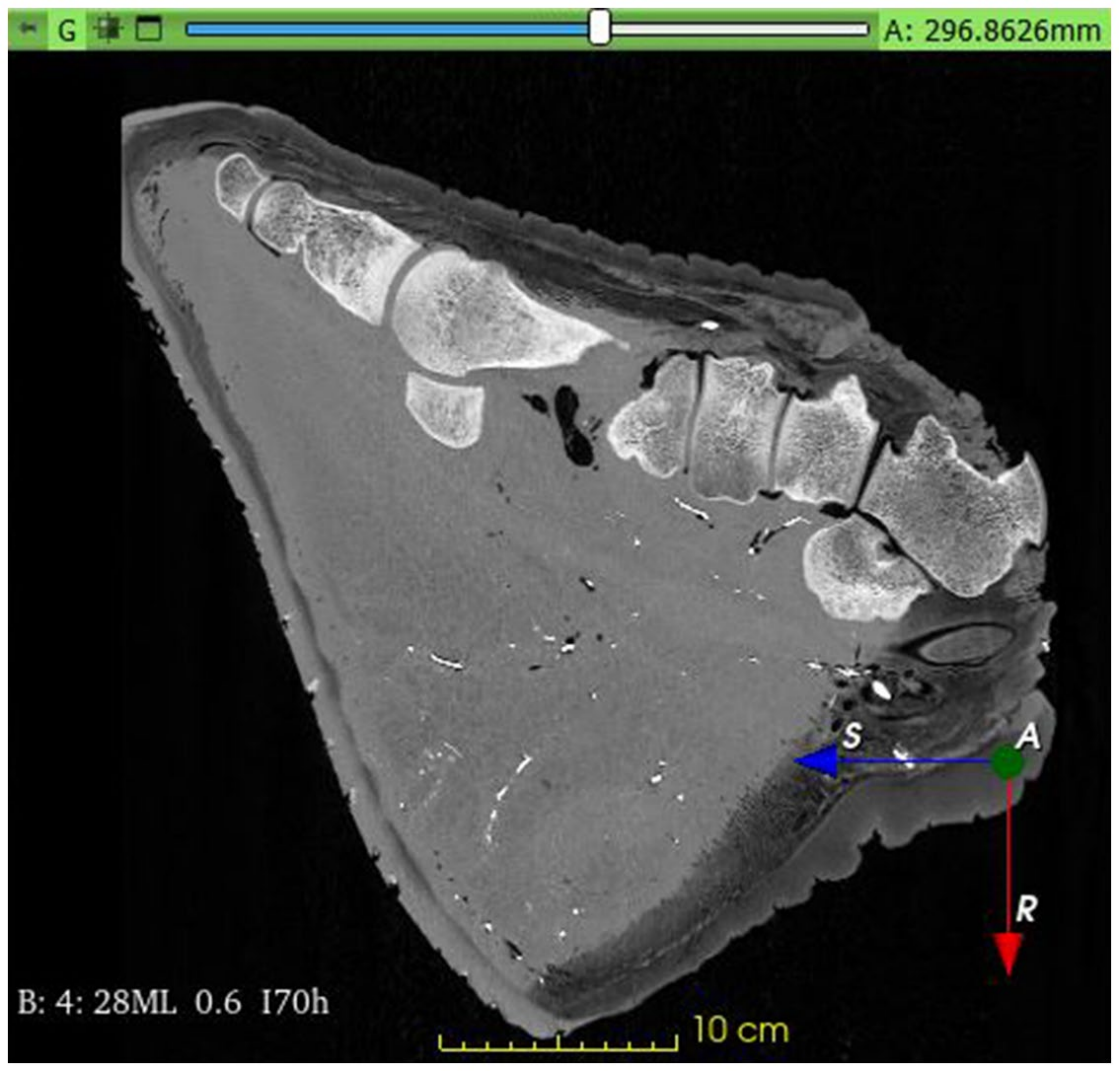
Contrast filled Computed Tomography image of the African savanna elephant’s right hindfoot. Mid-sagittal plane.

Following the native series, a total of 20 milliliters (ml) of barium sulfate-containing Micropaque contrast agent (Micropaque/Microtrast, Guerbet Inc., Villepinte, France) was administered over 3 min into the cannulated vessels, and a second series of CT scans was performed with similar settings as in the previous scan (Figure 1), directly after injecting the contrast agent. This was followed by an additional 3 min’ long injection of 28 mL contrast agent and a subsequent immediate third scan. Injection of further contrast agent became difficult at this point, and the process was ceased. The obtained material was saved in a DICOM format.

The sequences were evaluated by two veterinary surgeons with experience in elephant care and were found to be free of pathological lesions. The toenails possessed good shape and constitution which corresponded with the assessment that this elephant did not have foot-related disease. The skeletal maturation of the elephant was found to be complete, as the growth plates were completely closed. This observation was again in line with the documented age of the investigated animal, as this is a process typically occurring at the age of 9 to 10 years (29, 30).

The first series was selected to be used for the bone reconstruction process. The vessels in the 2nd series were not adequately filled, and the contrast agent did not reach the sole, but the vessels in the 3rd series were much more visible, so it was selected for reconstruction of the blood supply.

The chosen datasets were loaded into the open-source, free 3D Slicer software (31, 32). The current version at the time of use was version 5.2.2. The 3D Slicer is constantly being optimized with the help of the National Institutes of Health. The program for computerized image analysis is characterized by being freely available and not tied to specific hardware, which makes it easily accessible and user-friendly. It is intended for both medical and veterinary clinical research purposes in the realm of image processing, medical image informatics, and visualization using 3D models (31, 32).

**Figure 2:**
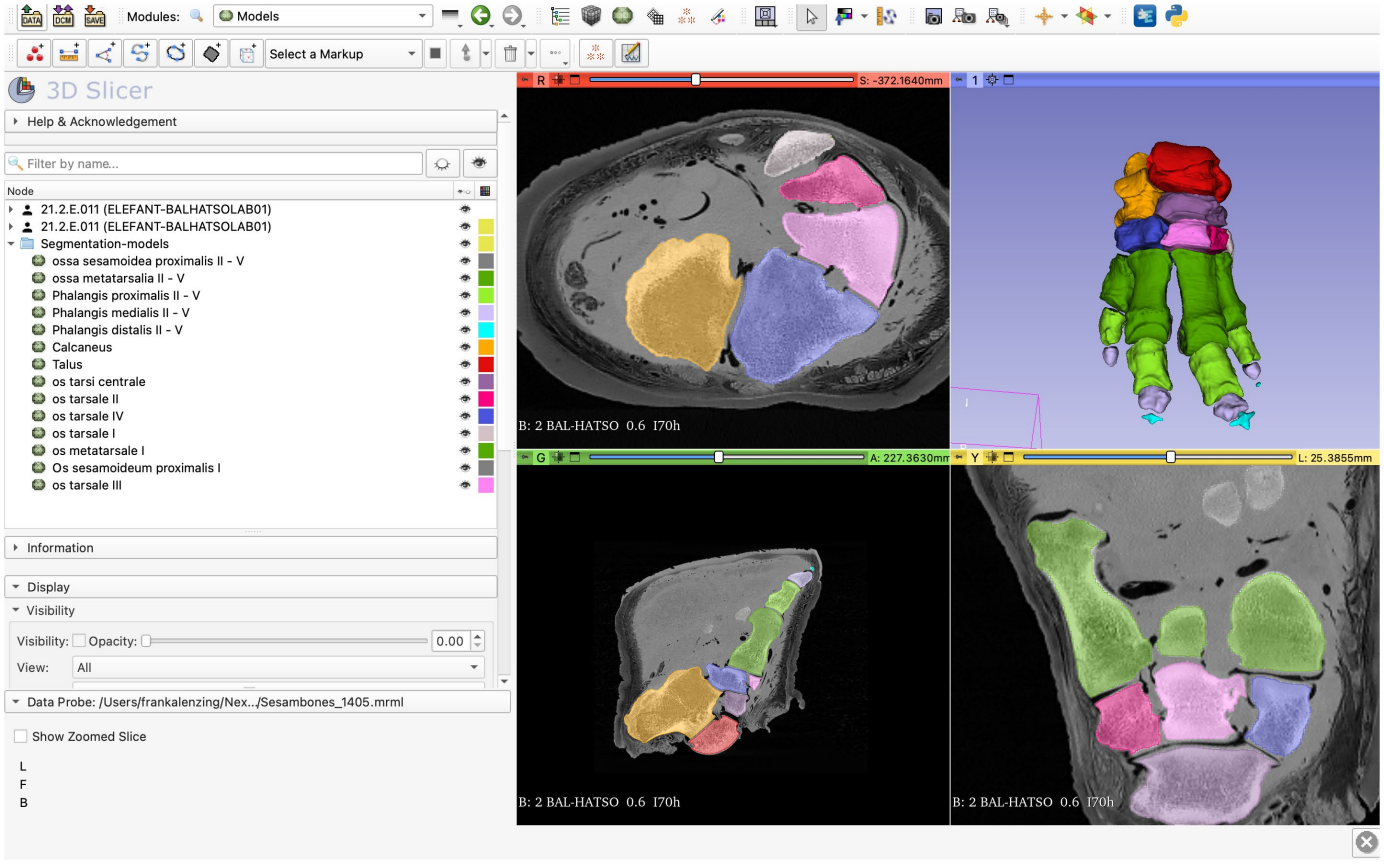
Screenshot of the 3D Slicer; completed process of transforming segments to models through the “Segment editor / Models” effect.

The “Paint” effect in the “Segment editor” module of the software was used to segment the bones. The voxels of each bone were manually stained one by one in the selected color to get clear borders and to visualize the different bone groups as thoroughly and realistically as possible (Figure 2). The software lacks a standard setting for each bone of the region and there is no generally accepted color code. Therefore, we selected colors from the available options that were readily distinguished from each other. Every bone group and every individual bone was colored in one slice. After finishing the coloration, the segments were converted to models through the “Segment editor / Models” effect to create a multicolored 3D reconstruction of the elephant’s foot.

The “Threshold” effect as an automatic segmentation, which can be found in the “Segment editor” module, was used for the vessel’s reconstruction. The “Threshold” effect identifies the density of the different tissues shown in the CT images. Through the “Threshold range,” the densities which will be colored can be determined (31, 32). For the vessels, the “Threshold range” was 2,800–3,070. This marked the basic structures of the main vessels without marking the other tissues. Afterwards an anatomist traced and verified every vessel and their smaller branches slice by slice and manually revised the segmentation using the “Segment editor / Paint” effect.

Once these models were saved, the “Threshold” effect was set to a wide range (99–3,071) on the same CT sequence, and every tissue was segmented into one block. This model was used to reconstruct the external view, the skin, and its features.

**Figure 3:**
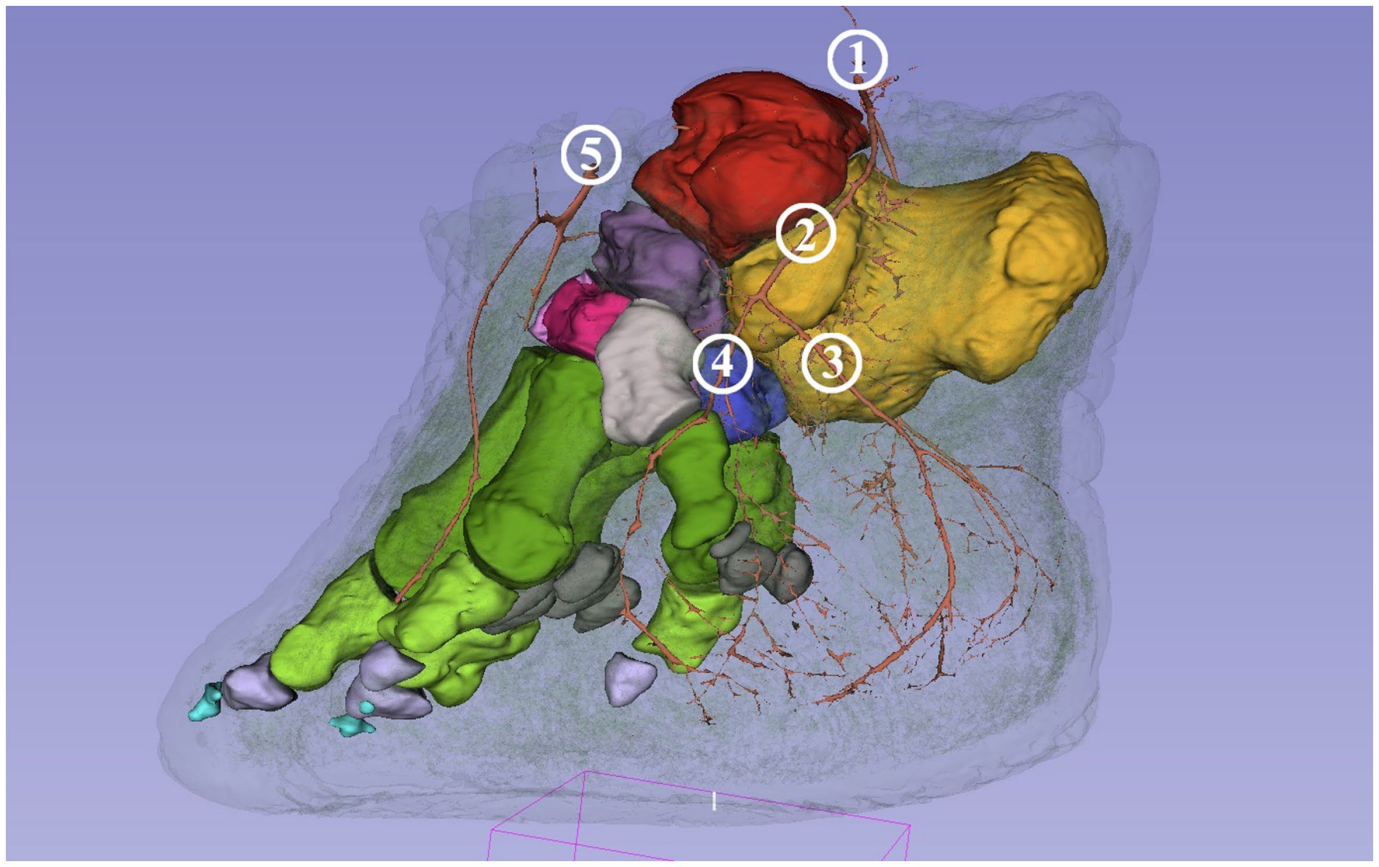
Screenshot of the 3D Slicer; composed 3D model of the African savanna elephant’s right hindfoot with bones and the arteries. The opacity of the neighboring soft tissue is reduced to 10%. Medial view. Red: talus, yellow: calcaneus, dark purple: os tali centrale, light gray: os tarsale I., violet: os tarsale II, light pink: os tarsale III, blue: os tarsale IV, dark green: ossa metatarsalia I-V, light green: phalanx proximalis I-V, light purple: phalanx media I-V, cyan:: phalanx distale I-V, dark gray: ossa sesamoidea proximalia, **1**: a. tibialis caudalis; **2**: a. plantaris medialis; **3**: a. plantaris medialis r. plantaris; **4**: a. plantaris medialis r. dorsalis; **5**: a. dorsalis pedis.

All models were merged to get the comprehensive connection between bones and arteries and to identify the arteries and branches in the following step. The vessels, individual bones, and skin features may be turned on and off or their opacity may be changed in order to get a better perspective (Figure 3).

## 3 Results

The finalized model enabled us to view a composite representation of all reconstructed tissues and examine the connection between bones, arteries, and skin. Identified vessels not previously described in the elephant are named according to the homologous vessels in the Illustrated Veterinary Anatomical Nomenclature (21).

The oval-shaped sole formed by mediolateral compression was seen, and the three toenails could be identified. The lack of pathological lesions in the foot indicates appropriate housing conditions and husbandry techniques. The reconstruction of the bones revealed comparatively small nutritional openings clustered on the *calcaneus* (especially on the *sustentaculum tali*), *talus,* and on the dorsal surface of the metatarsal bones. The plantar surface of the metatarsal bones remained smooth. The *prehallux* was visible in the CT images, but it was not stained together with the bones.

The detailed study of the 3D bone models confirmed that the *calcaneus* and the *talus* of African elephants form a *sinus tarsi.* The first digit had no phalanges, and the first metatarsus was the most distal bone, which was supported by a single proximal sesamoid bone at its distal epiphysis. The fifth digit lacked the distal phalange. All other digits had all three phalanges present with a pair of proximal sesamoid bones for each.

**Figure 4:**
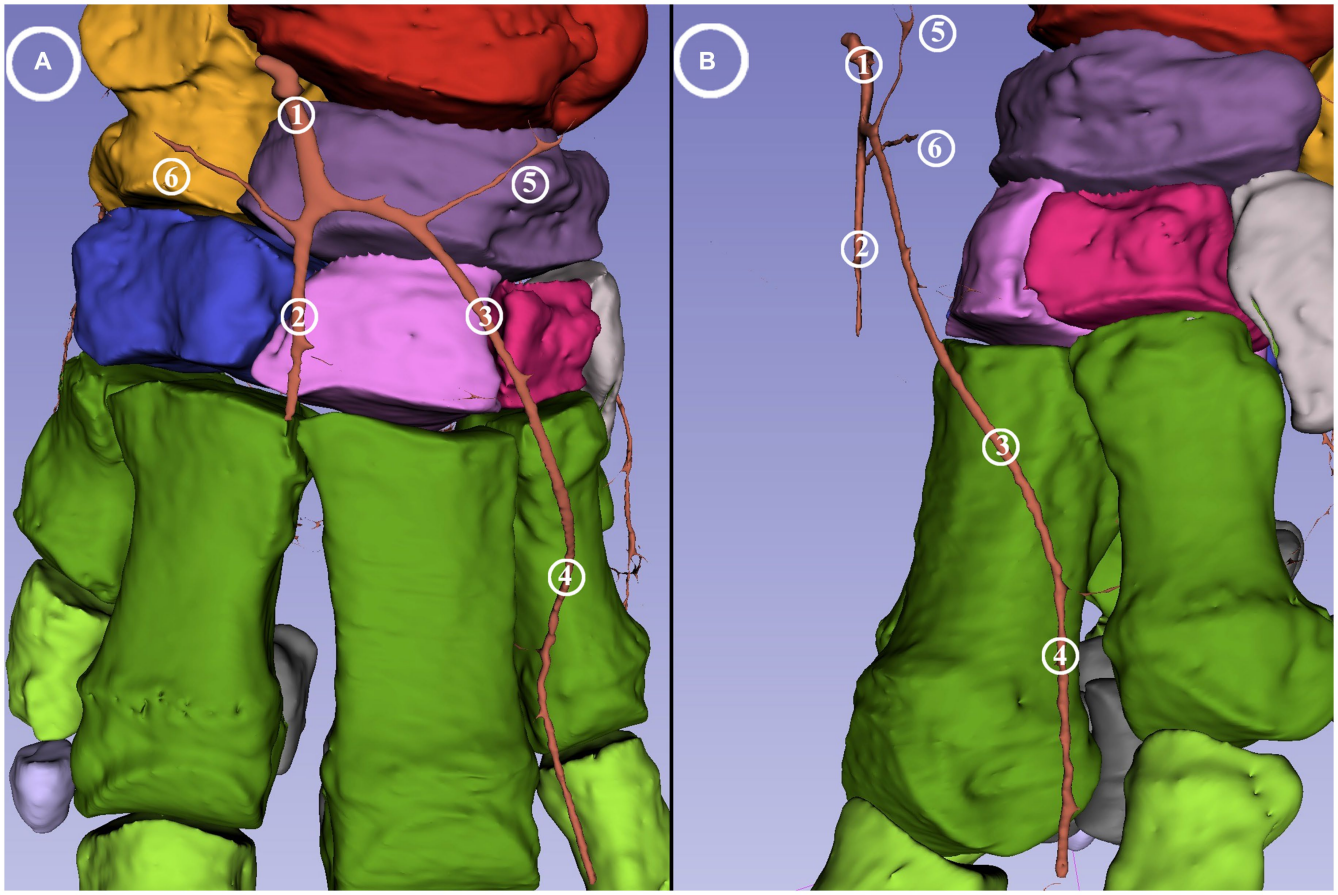
Dorsal blood supply of the African savanna elephant’s right hindfoot. Screenshot of the 3D Slicer. **(A)** Dorsal view. **(B)** Mediodorsal view. Red: talus, yellow: calcaneus, dark purple: os tali centrale, light gray: os tarsale I., violet: os tarsale II, light pink: os tarsale III, blue: os tarsale IV, dark green: ossa metatarsalia I-V, light green: phalanx proximalis I-V, light purple: phalanx media I-V, cyan:: phalanx distale I-V, dark gray: ossa sesamoidea proximalia, **1**: a. dorsalis pedis; **2**: a. digitalis dorsalis communis III; **3**: a. digitalis dorsalis communis II; **4**: a. digitalis dorsalis propria III abaxialis; **5**: a. tarsea medialis; **6**: a. tarsea lateralis.

On the dorsal aspect of the foot, we saw a descending *a. dorsalis pedis* which divided into two well-visible arteries. The *a. digitalis dorsalis communis II* ran in a mediodistal direction over the third tarsal bone and then between the second and the third metatarsal bones. It gave off *a. digitalis dorsalis propria III abaxialis*, which was only traceable to the first phalanx (Figure 4).

The *a. digitalis dorsalis communis III* ran distally over the dorsal aspect of the third tarsal bone and could be traced to the height of the basis of metatarsal bones three and four. From both common digital dorsal arteries, a branch (*a. tarsea lateralis et medialis*) was visible coursing in the abaxial direction of the respective side over the proximal row of the tarsus.

On the plantar aspect proximal to the *calcaneus,* the caudal branch of the caudal tibial artery divided into a medial (*a. plantaris medialis*) and a lateral branch (*a. plantaris lateralis*) which ran in a mediodistal and laterodistal direction, respectively.

The medial plantar artery gave rise to small branches running toward the nutritional holes on the caudomedial aspect of the *calcaneus*.

**Figure 5:**
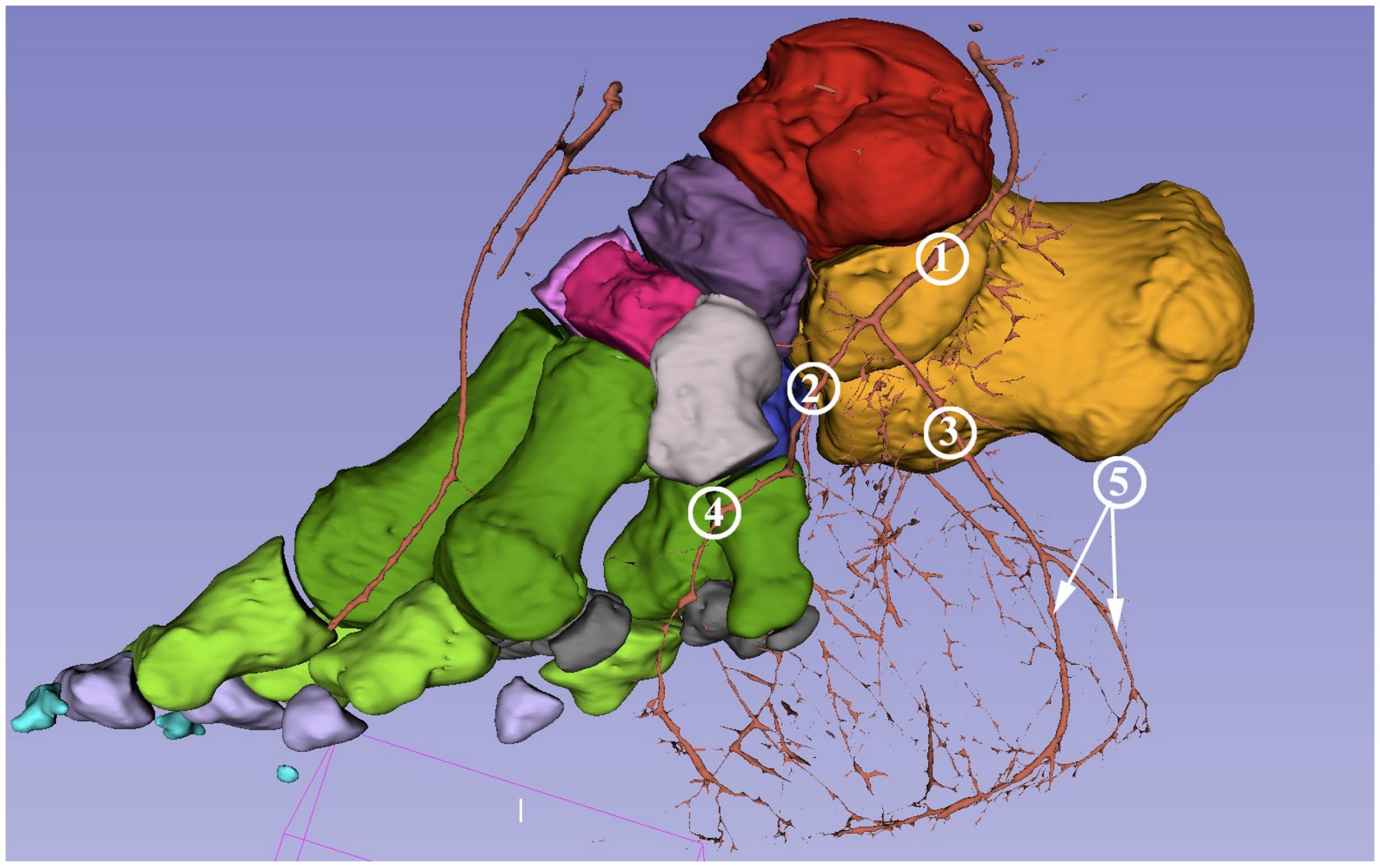
Blood supply of the digital cushion of the African savanna elephant’s right hindfoot. Screenshot of the 3D Slicer. Medial view. Red: talus, yellow: calcaneus, dark purple: os tali centrale, light gray: os tarsale I., violet: os tarsale II, light pink: os tarsale III, blue: os tarsale IV, dark green: ossa metatarsalia I-V, light green: phalanx proximalis I-V, light purple: phalanx media I-V, cyan:: phalanx distale I-V, dark gray: ossa sesamoidea proximalia, **1**: a. plantaris medialis; **2**: a. plantaris medialis r. dorsalis; **3**: a. plantaris medialis r. plantaris; **4**: a. digitalis dorsalis propria I abaxialis; **5**: rr. tori digitalis.

Both the lateral and the medial plantar arteries divided into a dorsal and a plantar branch (*ramus (r.) dorsalis et r. plantaris*). The *a. plantaris medialis* divided at the level of the central tarsal bone while the *a. plantaris lateralis* divided slightly more distally at the level of the fourth tarsal bone. The dorsal branch of the medial plantar artery vascularized the middle and distal row of the tarsus and ran on the medial aspect of the first metatarsal bone before giving off a medial branch to the dorsomedial side of the digital cushion. The remaining portion ran around the distal phalange of the first digit (*a. digitalis dorsalis propria I abaxialis*), descended until the sole, and finally turned in the plantar direction to supply the digital cushion. The plantar branch of both plantar arteries divided into a similarly strong lateral and medial branches (*rr. tori digitalis*) at level of the middle of the first phalanges. These supplied the digital cushion and the caudal portion of the sole from the medioplantar and lateroplantar directions, respectively (Figure 5); they bent toward the plantar direction, with the arch almost reaching the plantar skin and the terminal portion located 0.5 cm from the sole skin.

**Figure 6:**
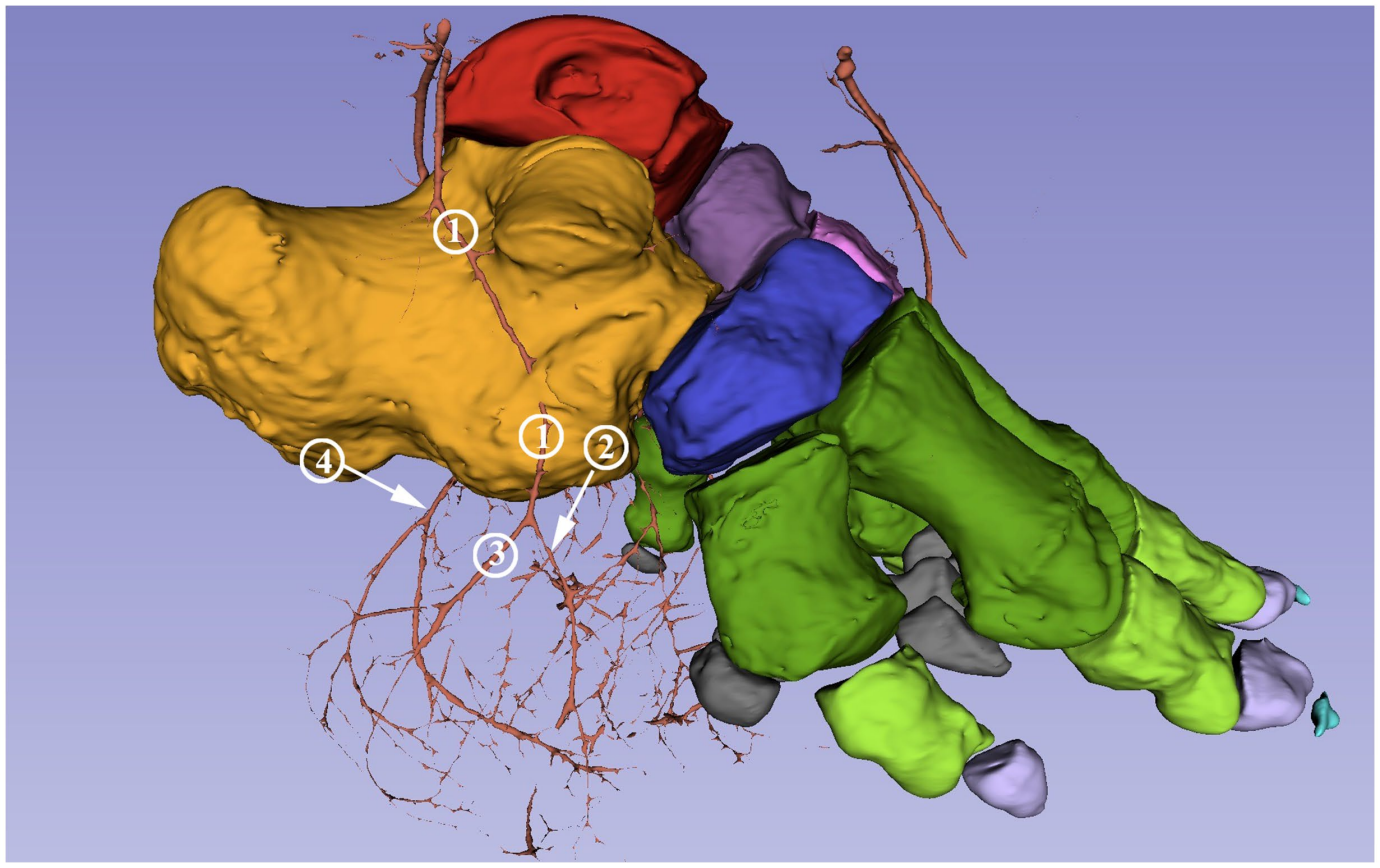
Blood supply of the digital cushion of the African savanna elephant’s right hindfoot. Screenshot of the 3D Slicer. Lateral view. Red: talus, yellow: calcaneus, dark purple: os tali centrale, light gray: os tarsale I., violet: os tarsale II, light pink: os tarsale III, blue: os tarsale IV, dark green: ossa metatarsalia I-V, light green: phalanx proximalis I-V, light purple: phalanx media I-V, cyan:: phalanx distale I-V, dark gray: ossa sesamoidea proximalia, **1**: a. plantaris lateralis; **2**: a. plantaris lateralis r. dorsalis; **3**: a. plantaris lateralis r. plantaris; **4**: a. plantaris medialis r. plantaris.

**Figure 7:**
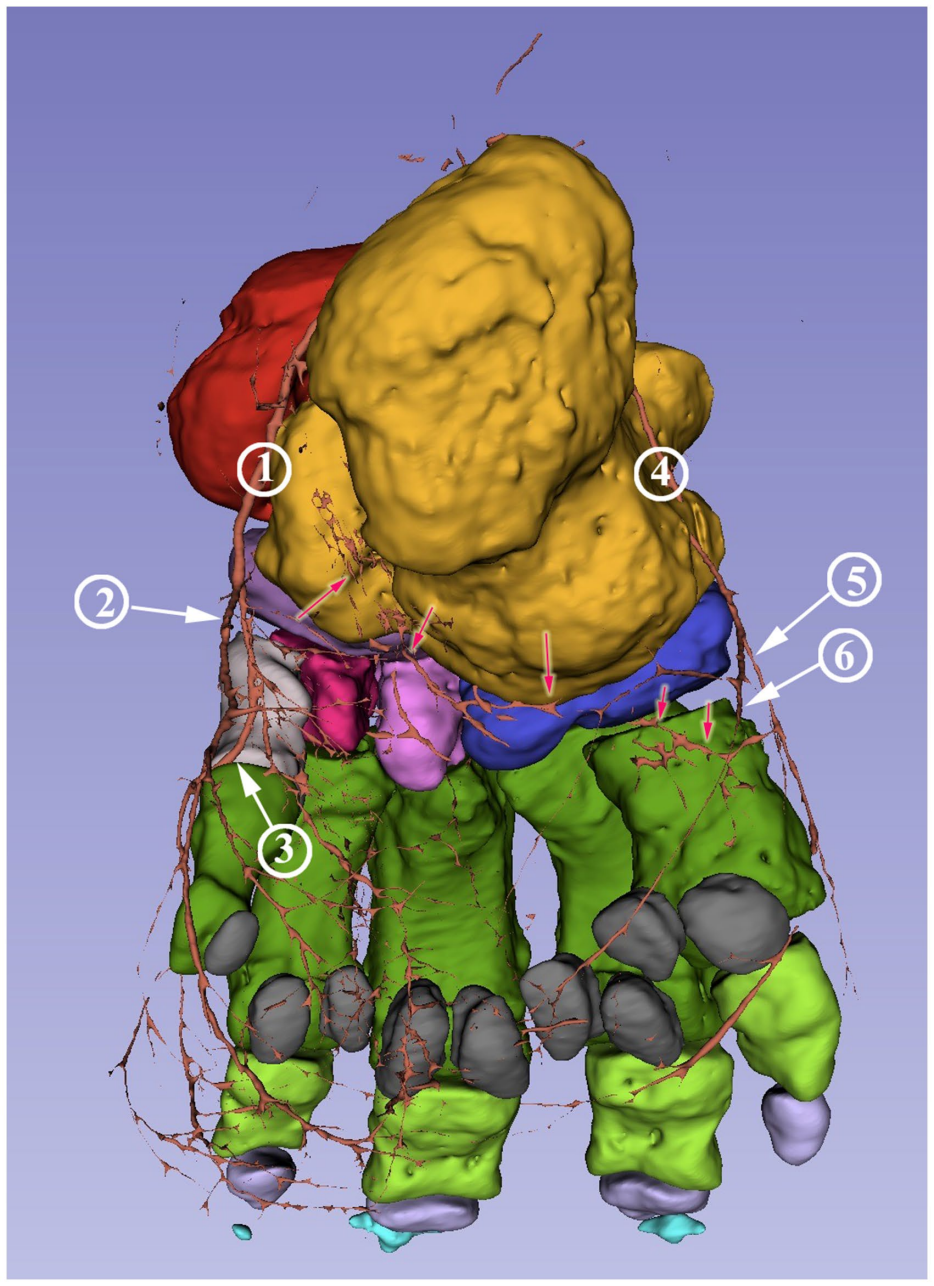
Blood supply of the African savanna elephant’s right hindfoot. Screenshot of the 3D Slicer. Plantar view. Red: talus, yellow: calcaneus, dark purple: os tali centrale, light gray: os tarsale I., violet: os tarsale II, light pink: os tarsale III, blue: os tarsale IV, dark green: ossa metatarsalia I-V, light green: phalanx proximalis I-V, light purple: phalanx media I-V, cyan:: phalanx distale I-V, dark gray: ossa sesamoidea proximalia, **1**: a. plantaris medialis; **2**: a. plantaris medialis r. plantaris; **3**: a. plantaris medialis r. dorsalis; **4**: a. plantaris lateralis; **5**: a. plantaris lateralis r. plantaris; **6**: a. plantaris lateralis r. dorsalis; the red arrows mark the visible portions of the arcus plantaris profundus.

The dorsal branch of the lateral plantar artery did not have any direct branch for the digits. Its main portion supplied the dorsolateral side of the digital cushion (Figure 6). It had a branch running horizontally in the medial direction behind the proximal epiphysis of the fifth metatarsal bone. This built an arch toward the medial plantar artery under the calcaneus, but the reconstruction is incomplete at this section (Figure 7). The plantar branch of the lateral plantar artery mirrored the path of the plantar branch of the medial plantar artery; thus, it supplied the digital cushion from a lateroplantar direction.

## 4 Discussion

The aim of this study was to identify and visualize the blood supply of the African elephant’s hindfoot and to present it in an approachable way. 3D models are highly beneficial for spatial representation as they are realistic and facilitate the imagination of complex structures.

The animal’s death was unexpected due to its rapidly progressive disease. The necessary pathological necropsy and post-mortem administrative paperwork, as well as coordination between the three institutes, took significant time. As a result, the team had to freeze the limb in order to preserve it.

Gross dissection, cryosectioning, and standard & CT angiography are alternative methods to obtain morphological data on the blood supply. Gross dissection and cryosectioning require a fresh cadaver however (33). Dissection of the specimen following the CT examinations would not have provided additional information. It is unlikely that tracing the branches that the contrast agent did not fill would have been feasible.

Basic angiography on this specimen would provide much less information than a CT and would not permit reconstruction. CT angiography (or positive contrast imaging) in a living elephant could lead to better results as the perfusion of the tissue would better distribute the contrast agent. However, as there is no literature on contrast imaging in live elephants, this option is likely precluded by the current technical limitations. CT scanners are located indoors in conditions that are inaccessible for adult elephants. While some specially trained dogs can remain motionless for the duration of a CT scan (34, 35) this is not realistic for elephants, whose slightest movement could seriously damage the equipment. Therefore, full anesthesia would be required; however current patient tables of the CT scanners cannot move the animal.

Reconstructive anatomical methods offer a more detailed assessment and significantly improve visualization compared to traditional techniques (36). Due to the change in permeability of the vascular wall, iodine-based contrast agents (such as those used in angiography) would rapidly penetrate into adjacent tissues. Although this technique is frequently used in certain studies (37) to increase tissue contrast, it would limit the visibility of the vessels that this study aimed to examine. No such effects of barium sulfate have been reported, even with an extended time period between injection and CT scans. However, during this period, due to its high viscosity, contrast can leak into small and insignificant vessels, leading to a decreased signal-to-noise ratio (38).

There are options in 3D Slicer to automate the segmentation process (31, 32), but that requires the subject to be in the exact same position for the different scans. During the injection of the contrast agent, the position of the limb was slightly altered, resulting in this method being inapplicable for our study. Therefore, we elected to proceed with manual segmentation.

This study is subject to certain limitations, mainly the utilization of only one specimen for the research. Anatomical studies of rare, endangered species often use only one (25, 39–42) or two (43) subjects. In addition, the possibility to use imaging techniques such as CT and MRI are also limited by the size, weight, and transport issues of this species (16) and the cost of such examinations. Greater collaboration among zoos would be necessary to include multiple healthy limbs in an extensive anatomical study.

No evidence of a correlation between body size or gender and foot morphology has been found in elephants (17, 19) or domesticated species (21, 23). Thus, individual anatomical differences, which would not have a clinical impact, may appear in our model, but the major structures are typically identical within the healthy members of the same species (21, 23). The vessels approaching the digital cushion are smaller in diameter then those supplying the digits, although the pad itself is much bigger in volume. They are well visible close to the skin but become scarce deeper. This supports the histological findings of Weissengruber et al. (18) and indicates limited healing capabilities for deep lesions.

Grouped nutritional holes were found on the *calcaneus*, *talus*, and dorsal surface of the metatarsal bones. Previously, nutritive openings were only described on the dorsal surface of the first phalangeal bones (17). Our study is the first to report the *sinus tarsi* in an African elephant. Although previously unreported in this species, it is in line with the anatomy of domestic mammals (20, 21) and the Asian elephant (25).

The filling of the specimen with contrast agent was stopped when the counterpressure became significant and the contrast agent started to appear on the cut surface of the limb. This indicated that the approachable vessels were full, and forcing more contrast agent could damage the arteries or create artifacts during the CT scans (38). Despite this, the reconstruction showed that the filling of the vessels was not complete. The plantar blood supply of the digits is not visible, either due to the collapse of the vessels during the process or blood clots already present at outset. Freezing could also have such an effect, but based on the authors’ experience with such specimens, the thawing time was sufficient to prevent residual frozen bodies from blocking the flow of the contrast agent. The visible vessels still provide valuable, novel information on the blood supply of the foot.

On the dorsal aspect of the foot in horses, cattle, sheeps & dogs, the *a. dorsalis pedis* descends until the distal third of the metatarsal bones where the dorsal common digital arteries would branch off, which make the *a. tarsea medialis et lateralis* well distinguishable branches of the *a. dorsalis pedis* at the height of the *art. centrodistalis* (23). In our study, the second and the third dorsal common digital arteries separate from the main vessel proximal to the distal row of the tarsus, making the medial and lateral tarsal arteries their subbranches on the respective sides. No further dorsal digital arteries were found. This can be the result of insufficient filling of the vessels, but in such cases, traces or short stumps are usually present (44). Therefore, it is more likely that digits I and V are fully supplied from the plantar direction just like the abaxial sides of digits II and IV.

On the plantar side the blood supply of the digital cushion is visible. Both the medial and lateral plantar arteries approach the pad from both the dorsal and the plantar directions and create many anastomoses close to the sole between the medial and lateral sides and the plantar and dorsal approaches as well. Ramsey & Henry did not report the division on the proximal portion of both the medial and the lateral plantar arteries or the anastomoses near the sole, indicating that their dissection stopped at the level of the tarsus (17). This setup is very different from the one described in the forelimb of the Asian elephant (25), where a single trunk arising from the *arcus palmaris profundus* would serve as the major vessel, and its direct branches reach the digital cushion close to the median plane of the limb. Previous studies state that the anatomy of the hindfoot in the different elephant species are similar (17, 18). Based on this, the blood supply to the digital cushion in the forelimb and hindlimb of elephants appears to differ significantly. This is a novel finding, as species with existing descriptions of this region, such as horses, cattle, and sheep, have similar blood supply to the digital cushion in each hoof. In those species the r*r. tori digitales* of the medial and lateral sides arise from the plantar digital arteries of the corresponding side on both the forelimb and the hindlimb (21, 23). However, the aforementioned studies about African elephants, as well as Asian elephants, focus on the locomotive apparatus rather than the vessels (17, 18). It is possible that the blood supply differs significantly despite the commonalities in the muscles and joints.

The incomplete arch connecting the medial and lateral plantar arteries distal to the calcaneus is probably the deep plantar arch (*arcus plantaris profundus*), which serves as the origin for the plantar blood supply of the digits in domestic mammals. As the arch itself is already fragmented, its branches are not distinguishable. The *a. tarsea perforans*, which runs through the canalis tarsi, was described in African savanna elephants (17) but we were unable to locate any sign of the vessel despite the presence of the canal. This could be the result of the insufficient filling of the contrast agent or an individual difference as this artery is reported to be inconstant in some species (21). For the contrast agent to sufficiently reach every major branch, including the mostly horizontally-oriented perforating tarsal artery and the deep plantar arch, a fresh carcass is needed, the limb should be severed more proximally and the blood should be flushed out before the contrast agent is injected.

## 5 Conclusion

The study provides novel information about the blood supply of the digital cushion in an African elephant. The previously described medial and lateral plantar arteries on each side of the tarsus divide into dorsal and plantar branches to supply the digital cushion and the caudal portion of the sole. The plantar branches are more dominant and running directly under the skin. The distribution of the arteries and their location compared to the skeleton and the skin are accessible through high quality images and 3D models, which are the first such to be presented of the region. The size and distribution of the vessels explain the slow healing of the digital cushion.

This anatomical description of the region will help caretakers and veterinarians execute treatment in the best possible way, which is especially important considering the vital role the condition of the foot and the digital cushion have in the general health of elephants.

While the forefeet and the hindfeet look very similar and have many similarities in their skeleton and dynamics, the blood supply of their digits and digital cushion are very different.

The 3D printable model can be used to educate students, caregivers, and veterinarians to help keep captive elephants healthy in the future, protecting the endangered species.

## Data availability statement

The datasets presented in this study can be found in online repositories. The names of the repository/repositories and accession number(s) can be found at: https://figshare.com/s/638488f9efbd1d75ede3.

## Ethics statement

Ethical approval was not required for the study involving animals in accordance with the local legislation and institutional requirements because we used the limb of a deceased elephant. The animal died due to natural causes before the study began. As the study only focuses on a limb of an already deceased animal, no ethical approval is necessary.

## Author contributions

LR: Conceptualization, Formal analysis, Funding acquisition, Investigation, Project administration, Software, Supervision, Writing – original draft, Writing – review & editing. FL: Formal analysis, Investigation, Software, Visualization, Writing – review & editing. EP: Investigation, Resources, Supervision, Validation, Writing – review & editing. AB: Resources, Supervision, Validation, Writing – review & editing. DF: Data curation, Resources, Visualization, Writing – review & editing. ÖP: Conceptualization, Investigation, Methodology, Resources, Software, Supervision, Visualization, Writing – review & editing.
